# Exact analytical soliton solutions of the M-fractional Akbota equation

**DOI:** 10.1038/s41598-024-64328-6

**Published:** 2024-06-11

**Authors:** Muath Awadalla, Aigul Taishiyeva, Ratbay Myrzakulov, Jihan Alahmadi, Abdullah A. Zaagan, Ahmet Bekir

**Affiliations:** 1 Department of Mathematics and Statistics, College of Science, King Faisal University, 31982 Hofuf, Al Ahsa Saudi Arabia; 2 Ratbay Myrzakulov Eurasian International Centre for Theoretical Physics, Astana, Kazakhstan; 3 Department of Mathematics, College of Science and Humanities in Al-Kharji, Prince Sattam Bin Abdulaziz University, 11942 Al-Kharj, Saudi Arabia; 4 Department of Mathematics, Faculty of Science, Jazan University, P.O. Box 2097, Jazan, 45142 Kingdom of Saudi Arabia; 5 Neighbourhood of Akcaglan, Imarli Street, Number: 28/4, 26030, Eskisehir, Turkey; 6 Kh.Dosmukhamedov, Atyrau University, Atyrau, Kazakhstan

**Keywords:** Fractional Akbota equation, $\exp _a$ function technique, Sardar sub-equation technique, Generalized Kudryashov technique, Analytical soliton solutions, Physics, Mathematics and computing, Applied mathematics

## Abstract

In this paper we explore the new analytical soliton solutions of the truncated M-fractional nonlinear $(1+1)$-dimensional Akbota equation by applying the $\exp _a$ function technique, Sardar sub-equation and generalized kudryashov techniques. Akbota is an integrable equation which is Heisenberg ferromagnetic type equation and have much importance for the analysis of curve as well as surface geometry, in optics and in magnets. The obtained results are in the form of dark, bright, periodic and other soliton solutions. The gained results are verified as well as represented by two-dimensional, three-dimensional and contour graphs. The gained results are newer than the existing results in the literature due to the use of fractional derivative. The obtained results are very helpful in optical fibers, optics, telecommunications and other fields. Hence, the gained solutions are fruitful in the future study for these models. The used techniques provide the different variety of solutions. At the end, the applied techniques are simple, fruitful and reliable to solve the other models in mathematical physics.

## Introduction

Fractional partial differential equations play fundamental role in physics as well as other branches of science like differential geometry of curves and surfaces. Many realistic models of phenomenons in different fields applications are represented in the form of nonlinear fractional partial differential equations (NFPDEs). Exploring the such NFPDEs is the very difficult work. However, there are some sector of NFPDEs which is called the integrable equations. These integrable NFPDEs can be solved by distinct techniques to obtain the exact analytical soliton solutions. For example; Unified technique^1^, modified direct algebraic technique^2^, generalized exponential rational function technique^3,4^, extended sinh-Gordon equation expansion technique^5,6^, the $(G'/G^2)$-expansion technique and many more^7–9^. It is found that some integrable equations are interrelated through the so-called gauge equivalences. One of such integrable system is an Akbota equation (AE) given as^10^:

1 $$\begin{aligned}{} & {} S_t-S\wedge (\theta _1 S_{xx}+\theta _2 S_{xt})-\theta _3 f S_{x}=0,\nonumber \\{} & {} f_x+S\times (S_x \wedge S_t)=0. \end{aligned}$$

along $S=(s_1,s_2,s_3)$ with $S^{2}=1$ and f is a scalar function while $\wedge$ represents the vector product.

The gauge equivalent counterpart of system.(1) is of the form given as^11^.

2 $$\begin{aligned}{} & {} \iota D_{M,t}^{\alpha ,\Upsilon }f+\theta _1 D_{M,xx}^{2\alpha ,\Upsilon }f+\theta _1 D_{M,xt}^{2\alpha ,\Upsilon }f+\theta _3 g f=0,\nonumber \\{} & {} D_{M,x}^{\alpha ,\Upsilon }g-2\varrho (\theta _1 D_{M,x}^{\alpha ,\Upsilon }|f|^{2}+\theta _2 D_{M,t}^{\alpha ,\Upsilon }|f|^{2})=0, \end{aligned}$$

where

3 $$\begin{aligned} D_{M,x}^{\alpha ,\Upsilon }f(x) = \mathop {\lim }\limits _{\tau \rightarrow 0} \frac{{f\,(x~{E_\Upsilon }(\tau {x^{1 - \alpha }})) - f(x)}}{\tau },\quad 0<\alpha \le 1,\;\;\Upsilon \in (0,\infty ) \end{aligned}$$

where ${E_\Upsilon }(.)$ is a truncated Mittag-Leffler (TML) function mention in^12,13^.

here $f=f(x,t)$ represents the complex-valued wave function while $g=g(x,t)$ shows the real valued wave profile. The symbols $\theta _i (i=1,2,3)$ are the arbitrary constants and $\varrho =\pm 1$. Akbota equation admits two significant reductions:

(i) If $\theta _2=0$, the nonlinear Schrödinger equation
(ii) If $\theta _1=0$, the Kuralay equation.

The Akbota equation as a Heisenberg ferromagnetic type equation has much importance for the study of nonlinear phenomenons in magnets, optics and the differential geometry of curves and surfaces. Breather wave, rogue wave and semi-rational solutions of the Akbota equation have been obtained by using the J-fold Darboux transformation^14^.

In this research paper, we explore the new analytical soliton solutions to the truncated M-fractional nonlinear (1+1)-dimensional Akbota equation. For our this purpose, we utilized the three methods; $\exp _a$ function method, Sardar sub-equation method and generalized Kudryashov medhod. These methods have many applications in the literature. For example, optical wave solutions of the cold bosonic atoms in the model of zig-zag optical lattice are gained by using the $\exp _a$ function technique in^15^. The Sardar sub-equation approach has led to the discovery of new solitons for the (2+1)-dimensional Sawada-Kotera (SK) equation^16^, optical wave solutions of Fokas-Lenells model are obtained by utilizing the Sardar sub-equation technique in^17^. Similarly, different types of sioliton solutions likely, kink, bell shape, dark, solitary wave solutions and other soliton solutions of Fokas-Lenells equation are gained by applying generalized Kudryashov technique^18^, solitary wave solutions of Westervelt model are gained by applying generalized Kudryashov technique^19^.

The motivation of this paper is to explain the effect of M-fractional derivative on the solutions of space-time fractional Akbota equation that are gained with the use of $\exp _a$ function technique, Sardar sub-equation technique and generalized Kudryashov technique. Significance of M-fractional derivative is that it fulfills the both properties of integer and fractional order derivatives. By our used schemes, we can observe some elementary relationship between FNLPDEs and others simple NLODEs. It has been found that with the use of simple schemes and solvable ODEs, different type of exact wav solutions of some complicated FNLPDEs can be easily obtained. These are three different techniques used for solving nonlinear partial differential equations. Both methods are proposed to find the vast categories of exact wave solutions. By using the truncated M-fractional derivative, we gain the solutions very close to the numerical solutions. Our obtained solutions are newer than the existing solutions because truncated M-fractional derivative and the used techniques are first time used for the concerned model. The effect of truncated M-fractional derivative is also shown by 2-D graphs.

Paper consists on the different sections; Sect. "Description of techniques": this is about the complete description of the utilized techniques. Section "Mathematical treatment of the governing model": this is about the mathematical treatments of our concerned model. Section "Analytical soliton solutions": this is about the applications of the techniques to obtain the exact analytical soliton solutions of our concerned model. Section "Graphically representation": this is about the graphically representation of some of the obtained solutions. Section "Results and discussion": this is about the results and discussion. Section "Conclusion": this is about the conclusion of our research work.

## Description of techniques

### $\exp _a$ function technique

Some of the main points of this method are given as.

Considering a nonlinear PDE;

4 $$\begin{aligned} S( h, h^{2}h_{x}, h_{t}, h_{xx}, h_{tt},h_{xt},...)=0. \end{aligned}$$

Equation (4) reduces into a nonlinear ODE:

5 $$\begin{aligned} T(H, H^{'}, H^{''},...,)=0. \end{aligned}$$

Applying the given wave transformations:

6 $$\begin{aligned} h(x,t)=H(\xi ),~~~~~~~~ \xi = \delta x+\lambda t. \end{aligned}$$

Assuming the results for Eq. (6) are:^20–23^:

7 $$\begin{aligned} H(\xi )=\frac{\alpha _0+\alpha _1 d^{\xi }+\text {...}+ \alpha _m d^{m \xi }}{\beta _0+\beta _1 d^{\xi }+... +\beta _m d^{m \xi } },~~d\ne 0,1. \end{aligned}$$

here $\alpha _j$ and $\beta _j (0\le j \le m )$ are the undetermined. Positive integer *m* is found by Homogenous balance approach into Eq. (5). Using Eq. (7) into Eq. (5), yields

8 $$\begin{aligned} \wp (d^\xi )=\ell _0+\ell _1 d^\xi +...+\ell _t d^{t\xi } =0,. \end{aligned}$$

Inserting $\ell _j~(0 \le j \le t)$ into Eq. (8) taking zeo, a set of equations is attained:

9 $$\begin{aligned} \ell _j=0, ~~~here ~~j=0,...,t. \end{aligned}$$

Applying obtain solutions, one can get exact solitons for Eq. (4).

### Sardar sub-equation technique

This technique^24^ by considering the nonlinear fractional PDE:

10 $$\begin{aligned} J(g,g_{x},g_{xx},g_{xt},g g_{tt},g_{xxt},...)=0, \end{aligned}$$

where $g=g(z,t)$ is a wave profile. Substituting a wave transformation of the form:

11 $$\begin{aligned} g(x,t)=G(\zeta ),\; \zeta =\lambda x+\mu t. \end{aligned}$$

yields the following form of NLODE:

12 $$\begin{aligned} Y(G,G'',G G'',G' G^{2},...)=0. \end{aligned}$$

Consider a solution of Eq. (12) in the form:

13 $$\begin{aligned} G(\zeta )=\sum _{i=0}^{m}b_i\psi ^i(\zeta ), \end{aligned}$$

where $\psi (\zeta )$ satisfies the ODE given by

14 $$\begin{aligned} \psi ^{'}(\zeta )=\sqrt{\sigma +\kappa \psi ^{2}(\zeta )+\psi ^{4}(\zeta )}, \end{aligned}$$

in which $\sigma$ and $\kappa$ are parameters.

Next, we proceed by first substituting Eqs. (13) and (14) into Eq. (12) and sum the $\psi ^i$ term. Then, we set the coefficients of similar powers equal to zero to deduce a system of equations in $b_i$, $\lambda$, and $\mu$. By manipulating this system, we can determined the unknowns parameters. Case 1: if $\kappa >0$ and $\sigma =0$, we have

15 $$\begin{aligned} \psi ^{\pm }_{1}= & {} \pm \sqrt{-\kappa r s} ~sech_{r s} (\sqrt{\kappa } ~\zeta ), \end{aligned}$$

16 $$\begin{aligned} \psi ^{\pm }_{2}= & {} \pm \sqrt{\kappa r s} ~{{\,\textrm{csch}\,}}_{r s} (\sqrt{\kappa } ~\zeta ), \end{aligned}$$

where, $sech_{r s}( \zeta )=\frac{2}{re^{\zeta }+se^{-\zeta }}$, ${{\,\textrm{csch}\,}}_{r s}( \zeta )=\frac{2}{re^{\zeta }-se^{-\zeta }}.$ Case 2: if $\kappa <0$ and $\sigma =0$, we have

17 $$\begin{aligned} \psi ^{\pm }_{3}= & {} \pm \sqrt{-\kappa r s } ~\sec _{r s} (\sqrt{-\kappa } ~\zeta ), \end{aligned}$$

18 $$\begin{aligned} \psi ^{\pm }_{4}= & {} \pm \sqrt{-\kappa r s} ~\csc _{r s} (\sqrt{-\kappa } ~\zeta ), \end{aligned}$$

where, $\sec _{r s}( \zeta )=\frac{2}{r e^{\iota \zeta }+se^{-\iota \zeta }}$, $\csc _{r s}( \zeta )=\frac{2 \iota }{re^{\iota \zeta }-se^{-\iota \zeta }}.$ Case 3: if $\kappa <0$ and $\sigma =\frac{\kappa ^{2}}{4}$, we have

19 $$\begin{aligned} \psi ^{\pm }_{5}= & {} \pm \sqrt{-\frac{\kappa }{2}} ~\tanh _{r s} \left( \sqrt{-\frac{\kappa }{2}} ~\zeta \right) , \end{aligned}$$

20 $$\begin{aligned} \psi ^{\pm }_{6}= & {} \pm \sqrt{-\frac{\kappa }{2}} ~\coth _{r s} \left( \sqrt{-\frac{\kappa }{2}} ~\zeta \right) , \end{aligned}$$

21 $$\begin{aligned} \psi ^{\pm }_{7}= & {} \pm \sqrt{-\frac{\kappa }{2}}\left( \tanh _{r s} \left( \sqrt{-2 \kappa } ~\zeta \right) \pm \iota \sqrt{r s} ~sech_{r s} \left( \sqrt{-2 \kappa } ~\zeta \right) \right) , \end{aligned}$$

22 $$\begin{aligned} \psi ^{\pm }_{8}= & {} \pm \sqrt{-\frac{\kappa }{2}}\left( \coth _{r s} \left( \sqrt{-2 \kappa } ~\zeta \right) \pm \sqrt{{r s}} ~csch_{r s} \left( \sqrt{-2 \kappa } ~\zeta \right) \right) , \end{aligned}$$

23 $$\begin{aligned} \psi ^{\pm }_{9}= & {} \pm \sqrt{-\frac{\kappa }{8}}\left( \tanh _{r s} \left( \sqrt{-\frac{\kappa }{8}} ~\zeta \right) + \coth _{r s} \left( \sqrt{-\frac{\kappa }{8}} ~\zeta \right) \right) , \end{aligned}$$

where, $$\tanh _{r s}( \zeta )=\frac{re^{ \zeta }-se^{- \zeta }}{re^{ \zeta }+se^{- \zeta }}$$, $$\coth _{r s}( \zeta )=\frac{re^{ \zeta }+se^{- \zeta }}{re^{ \zeta }-se^{- \zeta }}.$$

Case 4: if $\kappa >0$ and $\sigma =\frac{\kappa ^{2}}{4}$, we have

24 $$\begin{aligned} \psi ^{\pm }_{10}= & {} \pm \sqrt{\frac{\kappa }{2}} ~\tan _{r s} \left( \sqrt{\frac{\kappa }{2}} ~\zeta \right) , \end{aligned}$$

25 $$\begin{aligned} \psi ^{\pm }_{11}= & {} \pm \sqrt{\frac{\kappa }{2}} ~\cot _{r s} \left( \sqrt{\frac{\kappa }{2}} ~\zeta \right) , \end{aligned}$$

26 $$\begin{aligned} \psi ^{\pm }_{12}= & {} \pm \sqrt{\frac{\kappa }{2}}\left( \tan _{r s} (\sqrt{2 \kappa } ~\zeta )\pm \sqrt{r s} ~\sec _{r s} \left( \sqrt{2 \kappa } ~\zeta \right) \right) , \end{aligned}$$

27 $$\begin{aligned} \psi ^{\pm }_{13}= & {} \pm \sqrt{\frac{\kappa }{2}}\left( \cot _{r s} \left( \sqrt{2 \kappa } ~\zeta \right) \pm \sqrt{r s} ~\csc _{r s} \left( \sqrt{2 \kappa } ~\zeta \right) \right) , \end{aligned}$$

28 $$\begin{aligned} \psi ^{\pm }_{14}= & {} \pm \sqrt{\frac{\kappa }{8}}\left( \tan _{r s} \left( \sqrt{\frac{\kappa }{8}} ~\zeta \right) + \cot _{r s} \left( \sqrt{\frac{\kappa }{8}} ~\zeta \right) \right) , \end{aligned}$$

where, $$\tan _{r s}( \zeta )=-\iota \frac{re^{ \iota \zeta }-se^{- \iota \zeta }}{re^{\iota \zeta }+se^{-\iota \zeta }}$$, $$\cot _{r s}( \zeta )=\iota \frac{re^{ \iota \zeta }+se^{-\iota \zeta }}{re^{\iota \zeta }-se^{-\iota \zeta }}.$$

### The generalized Kudryashov technique

The main steps of this technique are given as^25,26^:

- *Step 1*
  Consider a nonlinear PDE:
  29 $$\begin{aligned} Y ( q, q^{2}q_{\gamma }, q_{\theta }, q_{\theta \theta }, q_{\gamma \gamma },q_{\gamma \theta },...)=0. \end{aligned}$$
  Supposing the below wave transformation:
  30 $$\begin{aligned} q(\gamma , ~\theta )=Q(\eta ), ~~~~~~\eta = \gamma -\nu \theta . \end{aligned}$$
  Here $\nu$ represents a parameter. By putting Eq. (30) into the Eq. (29), we get the following non-linear ODE given as:
  31 $$\begin{aligned} T(Q, Q^{'}, Q^{2}Q^{'}, Q^{''}, Q^{2} Q^{'},...)=0. \end{aligned}$$
- *Step 2*
  Consider the solutions of Eq. (31) are:
  32 $$\begin{aligned} Q(\eta )=\alpha _{0}+\sum _{j=1}^{m}\frac{\alpha _{j}}{(1+\psi (\eta ))^{j}}. \end{aligned}$$
  here $\alpha _{0}$ and $\alpha _{j}$, $(j=1, 2, 3,...,~ m)$ are unknowns. Consider $\psi$ is a new function of $\eta$ satisfy a ODE:
  33 $$\begin{aligned} \psi '(\eta )=\rho +\sigma \psi (\eta )+\Omega \psi ^{2}(\eta ). \end{aligned}$$
  where $\rho$, $\sigma$ and $\Omega$ represent the constants.
  Equation (33) has following solutions given in^27^:
  If all $\rho$, $\sigma$ and $\Omega$ are not equal to zero, we have
  34 $$\begin{aligned} \psi (\eta )= & {} \frac{1}{2 \Omega }\left( \sqrt{4 \rho \Omega -\sigma ^2} \tan \left( \frac{1}{2} \sqrt{4 \rho \Omega -\sigma ^2} \left( d_0+\eta \right) \right) -\sigma \right) , 4 \rho \Omega > \sigma ^2. \end{aligned}$$
  35 $$\begin{aligned} \psi (\eta )= & {} \frac{-1}{2 \Omega }\left( \sqrt{4 \rho \Omega -\sigma ^2} \cot \left( \frac{1}{2} \sqrt{4 \rho \Omega -\sigma ^2} \left( d_0+\eta \right) \right) + \sigma \right) , 4 \rho \Omega > \sigma ^2. \end{aligned}$$
  36 $$\begin{aligned} \psi (\eta )= & {} \frac{-1}{2 \Omega }\left( \sqrt{4 \rho \Omega -\sigma ^2} \tanh \left( \frac{1}{2} \sqrt{4 \rho \Omega -\sigma ^2} \left( d_0+\eta \right) \right) + \sigma \right) , 4 \rho \Omega < \sigma ^2. \end{aligned}$$
  37 $$\begin{aligned} \psi (\eta )= & {} \frac{-1}{2 \Omega }\left( \sqrt{4 \rho \Omega -\sigma ^2} \coth \left( \frac{1}{2} \sqrt{4 \rho \Omega -\sigma ^2} \left( d_0+\eta \right) \right) + \sigma \right) , 4 \rho \Omega < \sigma ^2. \end{aligned}$$
  38 $$\begin{aligned} \psi (\eta )= & {} \frac{-1}{ \Omega }\left( \frac{1}{d_{0}+\eta }+\frac{\sigma }{2}\right) , 4 \rho \Omega = \sigma ^2. \end{aligned}$$
  If $\rho =0$ and $\Omega \ne 0$, we have
  39 $$\begin{aligned} \psi (\eta )= & {} \frac{-1}{2 \Omega }\left( \sigma \tanh \left( \frac{\sigma }{2} \left( d_0+\eta \right) \right) + \sigma \right) , \sigma ^2>0. \end{aligned}$$
  40 $$\begin{aligned} \psi (\eta )= & {} \frac{-1}{2 \Omega }\left( \sigma \coth \left( \frac{\sigma }{2} \left( d_0+\eta \right) \right) + \sigma \right) , \sigma ^2>0. \end{aligned}$$
  41 $$\begin{aligned} \psi (\eta )= & {} \frac{1}{2 \Omega }\left( \sqrt{-\sigma ^{2}} \tan \left( \frac{\sqrt{-\sigma ^{2}}}{2} \left( d_0+\eta \right) \right) - \sigma \right) , \sigma ^2<0. \end{aligned}$$
  42 $$\begin{aligned} \psi (\eta )= & {} \frac{-1}{2 \Omega }\left( \sqrt{-\sigma ^{2}} \cot \left( \frac{\sqrt{-\sigma ^{2}}}{2} \left( d_0+\eta \right) \right) + \sigma \right) , \sigma ^2<0. \end{aligned}$$
  43 $$\begin{aligned} \psi (\eta )= & {} \frac{\sigma }{\sigma \exp (-\sigma (d_{0}+\eta ))-\Omega }, ~~~~~~~~~~\sigma \ne 0. \end{aligned}$$
  44 $$\begin{aligned} \psi (\eta )= & {} \frac{-1}{\Omega \eta }, ~~~~~~~~~~~\sigma =0. \end{aligned}$$
  If $\sigma =0$ and $\Omega \ne 0$, we have
  45 $$\begin{aligned} \psi (\eta )= & {} \frac{\sqrt{\rho \Omega }}{\Omega } ~\tan \left( \sqrt{\rho \Omega } \left( d_{0}+\eta \right) \right) , \rho \Omega >0. \end{aligned}$$
  46 $$\begin{aligned} \psi (\eta )= & {} -\frac{\sqrt{\rho \Omega }}{\Omega } ~\cot \left( \sqrt{\rho \Omega } \left( d_{0}+\eta \right) \right) , \rho \Omega >0. \end{aligned}$$
  47 $$\begin{aligned} \psi (\eta )= & {} -\frac{\sqrt{\rho \Omega }}{\Omega } ~\tanh \left( \sqrt{- \rho \Omega } \left( d_{0}+\eta \right) \right) , \rho \Omega <0. \end{aligned}$$
  48 $$\begin{aligned} \psi (\eta )= & {} -\frac{\sqrt{\rho \omega }}{\Omega } ~\coth \left( \sqrt{- \rho \Omega } \left( d_{0}+\eta \right) \right) , \rho \Omega <0. \end{aligned}$$
  49 $$\begin{aligned} \psi (\eta )= & {} \frac{-1}{\Omega (d_{0}+\eta )}, ~~~~~~~~~~~\rho =0. \end{aligned}$$
  If $\Omega =0$ and $\sigma \ne 0$, we have
  50 $$\begin{aligned} \psi (\eta )=\frac{1}{\sigma }\left( \exp \left( \sigma (d_{0}+\eta )\right) -\rho \right) . \end{aligned}$$
- *Step 3* Substituting Eq. 32) into Eq. 31) and summing up co-efficient of each power of $\psi (\eta )$. Putting the coefficients equal to 0, we achieve a set of algebraic equations containing $\alpha _{0}$ and $\alpha _{j}$, $(j=1, 2, 3,...,~ m)$ and parameters. Evaluating the gained set, we get the results for undetermined.
- *Step 4* Using the achieved results for $\alpha _{0}$ and $\alpha _{j}$, $(j=1, 2, 3,...,~ m)$ into Eq. 32) and using the Eqs. (34)–(50), we get the exact results for Eq. (29).

## Mathematical treatment of the governing model

Consider the travelling wave transformations:

51 $$\begin{aligned} f(x,t)=F(\xi )\times \exp (\iota \frac{\Gamma (1+\Upsilon )}{\alpha }(-a x^{\alpha }+\lambda t^{\alpha })),~~~~~~g(x,t)=G(\xi ), ~~~~~~\xi =\frac{\Gamma (1+\Upsilon )}{\alpha }(x^{\alpha }-\tau t^{\alpha }). \end{aligned}$$

By using the Eq. (51) into system (2), we attain the real and imaginary parts given as:

real part:

52 $$\begin{aligned} (-a^{2}\theta _1-\lambda +a \theta _2 \lambda +\theta _3 G)F+(\theta _1-\theta _2 \tau )F''=0. \end{aligned}$$

imaginary part:

53 $$\begin{aligned} (-2 a \theta _1-\tau +a \theta _2 \tau +\theta _2 \lambda )F'=0. \end{aligned}$$

54 $$\begin{aligned} -4 \varrho (\theta _1-\theta _2 \tau )FF'+G'=0. \end{aligned}$$

From Eq. (53), we get

55 $$\begin{aligned} \lambda =\frac{2 a \theta _1+\tau -a \theta _2 \tau }{\theta _2}. \end{aligned}$$

By integrating Eq. (54) w.r.t to $\xi$ and neglecting integration constant, we obtain

56 $$\begin{aligned} G(\xi )=2 \varrho (\theta _1-\theta _2 \tau ) F^{2}(\xi ). \end{aligned}$$

By putting Eqs. (56) and (55) into Eq. (52), we attain

57 $$\begin{aligned} \left( a \left( a \theta _2-2\right) \left( \theta _1-\theta _2 \tau \right) -\tau \right) F+2 \theta _2 \theta _3 \varrho \left( \theta _1-\theta _2 \tau \right) F^3+\theta _2 \left( \theta _1-\theta _2 \tau \right) F''=0. \end{aligned}$$

where $$\left( \theta _1-\theta _2 \tau \right) \ne 0$$.

By applying the Homogenous balance approach into Eq. (57), we gain the natural number $m=1$. Now we will solve the Eq. (57) with the help of above mentioned techniques.

## Analytical soliton solutions

### By $\exp _a$ function technique

Equation (7) transforms into given form for $m=1$:

58 $$\begin{aligned} H(\xi )=\frac{\alpha _0+\alpha _1 d^{\xi }}{\beta _0+\beta _1 d^{\xi }}. \end{aligned}$$

Using Eq. (58) into Eq. (57), we get distinct sets:

Set 1:

59 $$\begin{aligned}{} & {} \left\{ \alpha _0=\alpha _0,\alpha _1=\frac{i \beta _1 \sqrt{a \theta _1(a \theta _2-2)-\tau (a \theta _2-1){}^2}}{\sqrt{2 \theta _3 \theta _2 \varrho (\theta _1-\theta _2 \tau )}},\beta _0=-\frac{i \alpha _0 \sqrt{2 \theta _3 \theta _2 \varrho (\theta _1-\theta _2 \tau )}}{\sqrt{a \theta _1 (a \theta _2-2)-\tau (a \theta _2-1){}^2}},\beta _1=\beta _1\right\} . \end{aligned}$$

60 $$\begin{aligned}{} & {} f\left( x,t \right) =\frac{\sqrt{a \theta _1 \left( a \theta _2-2\right) -\tau \left( a \theta _2-1\right) {}^2} \left( \alpha _0 \sqrt{2 \theta _3 \theta _2 \varrho \left( \theta _1-\theta _2 \tau \right) }+i \beta _1 d^{\xi } \sqrt{a \theta _1 \left( a \theta _2-2\right) -\tau \left( a \theta _2-1\right) {}^2}\right) }{\sqrt{2 \theta _3 \theta _2 \varrho \left( \theta _1-\theta _2 \tau \right) } \left( \beta _1 d^{\left( \frac{\Gamma \left( 1+\Upsilon \right) }{\alpha }\left( x^{\alpha }-\tau t^{\alpha }\right) \right) } \sqrt{a \theta _1 \left( a \theta _2-2\right) -\tau \left( a \theta _2-1\right) {}^2}-i \alpha _0 \sqrt{2 \theta _3 \theta _2 \varrho \left( \theta _1-\theta _2 \tau \right) }\right) }\nonumber \\{} & {} \quad \times \exp \left( \iota \frac{\Gamma \left( 1+\Upsilon \right) }{\alpha }\left( -a x^{\alpha }+\left( \frac{2 a \theta _1+\tau -a \theta _2 \tau }{\theta _2}\right) t^{\alpha }\right) \right) . \end{aligned}$$

61 $$\begin{aligned}{} & {} g\left( x,t \right) =\left( \frac{\sqrt{a \theta _1 \left( a \theta _2-2\right) -\tau \left( a \theta _2-1\right) {}^2} \left( \alpha _0 \sqrt{2 \theta _3 \theta _2 \varrho \left( \theta _1-\theta _2 \tau \right) }+i \beta _1 d^{\xi } \sqrt{a \theta _1 \left( a \theta _2-2\right) -\tau \left( a \theta _2-1\right) {}^2}\right) }{ \sqrt{\theta _3 \theta _2} \left( \beta _1 d^{\left( \frac{\Gamma \left( 1+\Upsilon \right) }{\alpha }\left( x^{\alpha }-\tau t^{\alpha }\right) \right) } \sqrt{a \theta _1 \left( a \theta _2-2\right) -\tau \left( a \theta _2-1\right) {}^2}-i \alpha _0 \sqrt{2 \theta _3 \theta _2 \varrho \left( \theta _1-\theta _2 \tau \right) }\right) }\right) ^{2}. \end{aligned}$$

Set 2:

62 $$\begin{aligned}{} & {} \left\{ \alpha _0=\alpha _0,\alpha _1=-\frac{i \beta _1 \sqrt{a \theta _1 \left( a \theta _2-2\right) -\tau \left( a \theta _2-1\right) {}^2}}{\sqrt{2 \theta _3 \theta _2 \varrho \left( \theta _1-\theta _2 \tau \right) }},\beta _0=\frac{i \alpha _0 \sqrt{2 \theta _3 \theta _2 \varrho \left( \theta _1-\theta _2 \tau \right) }}{\sqrt{a \theta _1 \left( a \theta _2-2\right) -\tau \left( a \theta _2-1\right) {}^2}},\beta _1=\beta _1\right\} . \end{aligned}$$

63 $$\begin{aligned}{} & {} f\left( x,t\right) =\frac{\sqrt{a \theta _1 \left( a \theta _2-2\right) -\tau \left( a \theta _2-1\right) {}^2} \left( \alpha _0 \sqrt{2 \theta _3 \theta _2 \varrho \left( \theta _1-\theta _2 \tau \right) }-i \beta _1 d^{\xi } \sqrt{a \theta _1 \left( a \theta _2-2\right) -\tau \left( a \theta _2-1\right) {}^2}\right) }{\sqrt{2 \theta _3 \theta _2 \varrho \left( \theta _1-\theta _2\tau \right) } \left( \beta _1 d^{\xi } \sqrt{a \theta _1 \left( a \theta _2-2\right) -\tau \left( a \theta _2-1\right) {}^2}+i \alpha _0\sqrt{2 \theta _3\theta _2 \varrho \left( \theta _1-\theta _2 \tau \right) }\right) }\nonumber \\{} & {} \quad \times \exp \left( \iota \frac{\Gamma \left( 1+\Upsilon \right) }{\alpha }\left( -a x^{\alpha }+\left( \frac{2 a \theta _1+\tau -a \theta _2 \tau }{\theta _2}\right) t^{\alpha }\right) \right) . \end{aligned}$$

64 $$\begin{aligned}{} & {} g\left( x,t\right) =\left( \frac{\sqrt{a \theta _1 \left( a \theta _2-2\right) -\tau \left( a \theta _2-1\right) {}^2} \left( \alpha _0 \sqrt{2 \theta _3 \theta _2 \varrho \left( \theta _1-\theta _2 \tau \right) }-i \beta _1 d^{\xi } \sqrt{a \theta _1 \left( a \theta _2-2\right) -\tau \left( a \theta _2-1\right) {}^2}\right) }{\sqrt{ \theta _3 \theta _2 } \left( \beta _1 d^{\xi } \sqrt{a \theta _1 \left( a \theta _2-2\right) -\tau \left( a \theta _2-1\right) {}^2}+i \alpha _0\sqrt{2 \theta _3\theta _2 \varrho \left( \theta _1-\theta _2 \tau \right) }\right) }\right) ^{2}. \end{aligned}$$

where $$\xi =\frac{\Gamma (1+\Upsilon )}{\alpha }(x^{\alpha }-\tau t^{\alpha }).$$

### By Sardar sub-equation technique

For $m=1$, Eq. (13) reduces to the form:

65 $$\begin{aligned} F(\xi )=b_{0}+b_{1}\psi (\xi ). \end{aligned}$$

Inserting Eq. 65) into Eq. 57) by using Eq. 14), we obtain the solutionSet:

66 $$\begin{aligned} \left\{ b_0=0,b_1=\pm \frac{i}{\sqrt{\theta _3 \varrho }},\tau =\frac{\theta _1 \left( \theta _2 \left( a^2+\kappa \right) -2 a\right) }{\theta _2^2 \left( a^2+\kappa \right) -2 a \theta _2+1}\right\} . \end{aligned}$$

Case 1:

67 $$\begin{aligned} f\left( x,t\right)= & {} \pm \frac{i}{\sqrt{\theta _3 \varrho }} \left( \pm \sqrt{-\kappa r s} ~sech_{r s} \left( \sqrt{\kappa } ~\frac{\Gamma \left( 1+\Upsilon \right) }{\alpha }\left( x^{\alpha }-\tau t^{\alpha }\right) \right) \right) \nonumber \\{} & {} \times \exp \left( \iota \frac{\Gamma \left( 1+\Upsilon \right) }{\alpha }\left( -a x^{\alpha }+\left( \frac{2 a \theta _1+\tau -a \theta _2 \tau }{\theta _2}\right) t^{\alpha }\right) \right) . \end{aligned}$$

68 $$\begin{aligned} g\left( x,t\right)= & {} 2\left( \theta _1-\theta _2 \tau \right) \left( \frac{i}{\sqrt{\theta _3 }} \left( \sqrt{-\kappa r s} ~sech_{r s} \left( \sqrt{\kappa } ~\frac{\Gamma \left( 1+\Upsilon \right) }{\alpha }\left( x^{\alpha }-\tau t^{\alpha }\right) \right) \right) \right) ^{2}. \end{aligned}$$

69 $$\begin{aligned} f\left( x,t\right)= & {} \pm \frac{i}{\sqrt{\theta _3 \varrho }} \left( \pm \sqrt{\kappa r s} ~{{\,\textrm{csch}\,}}_{r s} \left( \sqrt{\kappa } ~\frac{\Gamma \left( 1+\Upsilon \right) }{\alpha }\left( x^{\alpha }-\tau t^{\alpha }\right) \right) \right) \nonumber \\{} & {} \times \exp \left( \iota \frac{\Gamma \left( 1+\Upsilon \right) }{\alpha }\left( -a x^{\alpha }+\left( \frac{2 a \theta _1+\tau -a \theta _2 \tau }{\theta _2}\right) t^{\alpha }\right) \right) . \end{aligned}$$

70 $$\begin{aligned} g\left( x,t\right)= & {} 2\left( \theta _1-\theta _2 \tau \right) \left( \frac{i}{\sqrt{\theta _3 }} \left( \sqrt{\kappa r s} ~{{\,\textrm{csch}\,}}_{r s} \left( \sqrt{\kappa } ~\frac{\Gamma \left( 1+\Upsilon \right) }{\alpha }\left( x^{\alpha }-\tau t^{\alpha }\right) \right) \right) \right) ^{2}. \end{aligned}$$

Case 2:

71 $$\begin{aligned} f\left( x,t\right)= & {} \pm \frac{i}{\sqrt{\theta _3 \varrho }} \left( \pm \sqrt{-\kappa r s } ~\sec _{r s} \left( \sqrt{-\kappa } ~\frac{\Gamma \left( 1+\Upsilon \right) }{\alpha }\left( x^{\alpha }-\tau t^{\alpha }\right) \right) \right) \nonumber \\{} & {} \times \exp \left( \iota \frac{\Gamma \left( 1+\Upsilon \right) }{\alpha }\left( -a x^{\alpha }+\left( \frac{2 a \theta _1+\tau -a \theta _2 \tau }{\theta _2}\right) t^{\alpha }\right) \right) . \end{aligned}$$

72 $$\begin{aligned} g\left( x,t\right)= & {} 2\left( \theta _1-\theta _2 \tau \right) \left( \frac{i}{\sqrt{\theta _3 }} \left( \sqrt{-\kappa r s } ~\sec _{r s} \left( \sqrt{-\kappa } ~\frac{\Gamma \left( 1+\Upsilon \right) }{\alpha }\left( x^{\alpha }-\tau t^{\alpha }\right) \right) \right) \right) ^{2}. \end{aligned}$$

73 $$\begin{aligned} f\left( x,t\right)= & {} \pm \frac{i}{\sqrt{\theta _3 \varrho }} \left( \pm \sqrt{-\kappa r s} ~\csc _{r s} \left( \sqrt{-\kappa } ~\frac{\Gamma \left( 1+\Upsilon \right) }{\alpha }\left( x^{\alpha }-\tau t^{\alpha }\right) \right) \right) \nonumber \\{} & {} \times \exp \left( \iota \frac{\Gamma \left( 1+\Upsilon \right) }{\alpha }\left( -a x^{\alpha }+\left( \frac{2 a \theta _1+\tau -a \theta _2 \tau }{\theta _2}\right) t^{\alpha }\right) \right) . \end{aligned}$$

74 $$\begin{aligned} g\left( x,t\right)= & {} 2\left( \theta _1-\theta _2 \tau \right) \left( \frac{i}{\sqrt{\theta _3 }} \left( \sqrt{-\kappa r s} ~\csc _{r s} \left( \sqrt{-\kappa } ~\frac{\Gamma \left( 1+\Upsilon \right) }{\alpha }\left( x^{\alpha }-\tau t^{\alpha }\right) \right) \right) \right) ^{2}. \end{aligned}$$

Case 3:

75 $$\begin{aligned} f\left( x,t\right)= & {} \pm \frac{i}{\sqrt{\theta _3 \varrho }} \left( \pm \sqrt{-\frac{\kappa }{2}} ~\tanh _{r s} \left( \sqrt{-\frac{\kappa }{2}} ~\frac{\Gamma \left( 1+\Upsilon \right) }{\alpha }\left( x^{\alpha }-\tau t^{\alpha }\right) \right) \right) \nonumber \\{} & {} \times \exp \left( \iota \frac{\Gamma \left( 1+\Upsilon \right) }{\alpha }\left( -a x^{\alpha }+\left( \frac{2 a \theta _1+\tau -a \theta _2 \tau }{\theta _2}\right) t^{\alpha }\right) \right) . \end{aligned}$$

76 $$\begin{aligned} g\left( x,t \right)= & {} 2\left( \theta _1-\theta _2 \tau \right) \left( \frac{i}{\sqrt{\theta _3 }} \left( \sqrt{-\frac{\kappa }{2}} ~\tanh _{r s} \left( \sqrt{-\frac{\kappa }{2}} ~\frac{\Gamma \left( 1+\Upsilon \right) }{\alpha }\left( x^{\alpha }-\tau t^{\alpha }\right) \right) \right) \right) ^{2}. \end{aligned}$$

77 $$\begin{aligned} f\left( x,t\right)= & {} \pm \frac{i}{\sqrt{\theta _3 \varrho }} \left( \pm \sqrt{-\frac{\kappa }{2}} ~\coth _{r s} \left( \sqrt{-\frac{\kappa }{2}} ~\frac{\Gamma \left( 1+\Upsilon \right) }{\alpha }\left( x^{\alpha }-\tau t^{\alpha }\right) \right) \right) \nonumber \\{} & {} \times \exp \left( \iota \frac{\Gamma \left( 1+\Upsilon \right) }{\alpha }\left( -a x^{\alpha }+\left( \frac{2 a \theta _1+\tau -a \theta _2 \tau }{\theta _2}\right) t^{\alpha }\right) \right) . \end{aligned}$$

78 $$\begin{aligned} g\left( x,t\right)= & {} 2\left( \theta _1-\theta _2 \tau \right) \left( \frac{i}{\sqrt{\theta _3 }} \left( \sqrt{-\frac{\kappa }{2}} ~\coth _{r s} \left( \sqrt{-\frac{\kappa }{2}} ~\frac{\Gamma \left( 1+\Upsilon \right) }{\alpha }\left( x^{\alpha }-\tau t^{\alpha }\right) \right) \right) \right) ^{2}. \end{aligned}$$

79 $$\begin{aligned} f\left( x,t\right)= & {} \pm \frac{i}{\sqrt{\theta _3 \varrho }} \left( \pm \sqrt{-\frac{\kappa }{2}}\left( \tanh _{r s} \left( \sqrt{-2 \kappa } ~\frac{\Gamma \left( 1+\Upsilon \right) }{\alpha }\left( x^{\alpha }-\tau t^{\alpha }\right) \right) \right. \right. \nonumber \\{} & {} \left. \left. \pm \iota \sqrt{r s} ~sech_{r s} \left( \sqrt{-2 \kappa } ~\frac{\Gamma \left( 1+\Upsilon \right) }{\alpha }\left( x^{\alpha }-\tau t^{\alpha }\right) \right) \right) \right) \nonumber \\{} & {} \times \exp \left( \iota \frac{\Gamma \left( 1+\Upsilon \right) }{\alpha }\left( -a x^{\alpha }+\left( \frac{2 a \theta _1+\tau -a \theta _2 \tau }{\theta _2}\right) t^{\alpha }\right) \right) . \end{aligned}$$

80 $$\begin{aligned} g\left( x,t\right)= & {} \frac{-2\left( \theta _1-\theta _2 \tau \right) }{\theta _3 } \left( \sqrt{-\frac{\kappa }{2}}\left( \tanh _{r s} \left( \sqrt{-2 \kappa } ~\frac{\Gamma \left( 1+\Upsilon \right) }{\alpha }\left( x^{\alpha }-\tau t^{\alpha }\right) \right) \right. \right. \nonumber \\{} & {} \left. \left. \pm \iota \sqrt{r s} ~sech_{r s} \left( \sqrt{-2 \kappa } ~\frac{\Gamma \left( 1+\Upsilon \right) }{\alpha }\left( x^{\alpha }-\tau t^{\alpha }\right) \right) \right) \right) ^{2}. \end{aligned}$$

81 $$\begin{aligned} f\left( x,t\right)= & {} \pm \frac{i}{\sqrt{\theta _3 \varrho }} \left( \pm \sqrt{-\frac{\kappa }{2}}\left( \coth _{r s} \left( \sqrt{-2 \kappa } ~\frac{\Gamma \left( 1+\Upsilon \right) }{\alpha }\left( x^{\alpha }-\tau t^{\alpha }\right) \right) \right. \right. \nonumber \\{} & {} \left. \left. \pm \sqrt{r s} ~csch_{r s} \left( \sqrt{-2 \kappa } ~\frac{\Gamma \left( 1+\Upsilon \right) }{\alpha }\left( x^{\alpha }-\tau t^{\alpha }\right) \right) \right) \right) \nonumber \\{} & {} \times \exp \left( \iota \frac{\Gamma \left( 1+\Upsilon \right) }{\alpha }\left( -a x^{\alpha }+\left( \frac{2 a \theta _1+\tau -a \theta _2 \tau }{\theta _2}\right) t^{\alpha }\right) \right) . \end{aligned}$$

82 $$\begin{aligned} g\left( x,t \right)= & {} \frac{-2\left( \theta _1-\theta _2 \tau \right) }{\theta _3 } \left( \sqrt{-\frac{\kappa }{2}}\left( \coth _{r s} \left( \sqrt{-2 \kappa } ~\frac{\Gamma \left( 1+\Upsilon \right) }{\alpha }\left( x^{\alpha }-\tau t^{\alpha }\right) \right) \right. \right. \nonumber \\{} & {} \left. \left. \pm \sqrt{r s} ~csch_{r s} \left( \sqrt{-2 \kappa } 
~\frac{\Gamma \left( 1+\Upsilon \right) }{\alpha }\left( x^{\alpha }-\tau t^{\alpha }\right) \right) \right) \right) ^{2}. \end{aligned}$$

83 $$\begin{aligned} f\left( x,t\right)= & {} \pm \frac{i}{\sqrt{\theta _3 \varrho }} \left( \pm \sqrt{-\frac{\kappa }{8}}\left( \tanh _{r s} \left( \sqrt{-\frac{\kappa }{8}} ~\frac{\Gamma \left( 1+\Upsilon \right) }{\alpha }\left( x^{\alpha }-\tau t^{\alpha }\right) \right) \right. \right. \nonumber \\{} & {} \left. \left. + \coth _{r s} \left( \sqrt{-\frac{\kappa }{8}} ~\frac{\Gamma \left( 1+\Upsilon \right) }{\alpha }\left( x^{\alpha }-\tau t^{\alpha }\right) \right) \right) \right) \nonumber \\{} & {} \times \exp \left( \iota \frac{\Gamma \left( 1+\Upsilon \right) }{\alpha }\left( -a x^{\alpha }+\left( \frac{2 a \theta _1+\tau -a \theta _2 \tau }{\theta _2}\right) t^{\alpha }\right) \right) . \end{aligned}$$

84 $$\begin{aligned} g\left( x,t \right)= & {} 2\left( \theta _1-\theta _2 \tau \right) \left( \frac{i}{\sqrt{\theta _3 }} \left( \sqrt{-\frac{\kappa }{8}}\left( \tanh _{r s} \left( \sqrt{-\frac{\kappa }{8}} ~\frac{\Gamma \left( 1+\Upsilon \right) }{\alpha }\left( x^{\alpha }-\tau t^{\alpha }\right) \right) \right. \right. \right. \nonumber \\{} & {} \left. \left. \left. + \coth _{r s} \left( \sqrt{-\frac{\kappa }{8}} ~\frac{\Gamma \left( 1+\Upsilon \right) }{\alpha }\left( x^{\alpha }-\tau t^{\alpha }\right) \right) \right) \right) \right) ^{2}. \end{aligned}$$

Case 4:

85 $$\begin{aligned} f\left( x,t\right)= & {} \pm \frac{i}{\sqrt{\theta _3 \varrho }} \left( \pm \sqrt{\frac{\kappa }{2}} ~\tan _{r s} \left( \sqrt{\frac{\kappa }{2}} ~\frac{\Gamma \left( 1+\Upsilon \right) }{\alpha }\left( x^{\alpha }-\tau t^{\alpha }\right) \right) \right) \nonumber \\{} & {} \times \exp \left( \iota \frac{\Gamma \left( 1+\Upsilon \right) }{\alpha }\left( -a x^{\alpha }+\left( \frac{2 a \theta _1+\tau -a \theta _2 \tau }{\theta _2}\right) t^{\alpha }\right) \right) . \end{aligned}$$

86 $$\begin{aligned} g\left( x,t\right)= & {} 2\left( \theta _1-\theta _2 \tau \right) \left( \frac{i}{\sqrt{\theta _3 }} \left( \sqrt{\frac{\kappa }{2}} ~\tan _{r s} \left( \sqrt{\frac{\kappa }{2}} ~\frac{\Gamma \left( 1+\Upsilon \right) }{\alpha }\left( x^{\alpha }-\tau t^{\alpha }\right) \right) \right) \right) ^{2}. \end{aligned}$$

87 $$\begin{aligned} f\left( x,t \right)= & {} \pm \frac{i}{\sqrt{\theta _3 \varrho }} \left( \pm \sqrt{\frac{\kappa }{2}} ~\cot _{r s} \left( \sqrt{\frac{\kappa }{2}} ~\frac{\Gamma \left( 1+\Upsilon \right) }{\alpha }\left( x^{\alpha }-\tau t^{\alpha }\right) \right) \right) \nonumber \\{} & {} \times \exp \left( \iota \frac{\Gamma \left( 1+\Upsilon \right) }{\alpha }\left( -a x^{\alpha }+\left( \frac{2 a \theta _1+\tau -a \theta _2 \tau }{\theta _2}\right) t^{\alpha }\right) \right) . \end{aligned}$$

88 $$\begin{aligned} g\left( x,t \right)= & {} 2\left( \theta _1-\theta _2 \tau \right) \left( \frac{i}{\sqrt{\theta _3 }} \left( \sqrt{\frac{\kappa }{2}} ~\cot _{r s} \left( \sqrt{\frac{\kappa }{2}} ~\frac{\Gamma \left( 1+\Upsilon \right) }{\alpha }\left( x^{\alpha }-\tau t^{\alpha }\right) \right) \right) \right) ^{2}. \end{aligned}$$

89 $$\begin{aligned} f\left( x,t \right)= & {} \pm \frac{i}{\sqrt{\theta _3 \varrho }} \left( \pm \sqrt{\frac{\kappa }{2}}\left( \tan _{r s} \left( \sqrt{2 \kappa } ~\frac{\Gamma \left( 1+\Upsilon \right) }{\alpha }\left( x^{\alpha }-\tau t^{\alpha }\right) \right) \right. \right. \nonumber \\{} & {} \left. \left. \pm \sqrt{r s} ~\sec _{r s} \left( \sqrt{2 \kappa } ~\frac{\Gamma \left( 1+\Upsilon \right) }{\alpha }\left( x^{\alpha }-\tau t^{\alpha }\right) \right) \right) \right) \nonumber \\{} & {} \times \exp \left( \iota \frac{\Gamma \left( 1+\Upsilon \right) }{\alpha }\left( -a x^{\alpha }+\left( \frac{2 a \theta _1+\tau -a \theta _2 \tau }{\theta _2}\right) t^{\alpha }\right) \right) . \end{aligned}$$

90 $$\begin{aligned} g\left( x,t \right)= & {} 2\left( \theta _1-\theta _2 \tau \right) \left( \frac{i}{\sqrt{\theta _3 }} \left( \sqrt{\frac{\kappa }{2}}\left( \tan _{r s} \left( \sqrt{2 \kappa } ~\frac{\Gamma \left( 1+\Upsilon \right) }{\alpha }\left( x^{\alpha }-\tau t^{\alpha }\right) \right) \right. \right. \right. \nonumber \\{} & {} \left. \left. \left. \pm \sqrt{r s} ~\sec _{r s} \left( \sqrt{2 \kappa } ~\frac{\Gamma \left( 1+\Upsilon \right) }{\alpha }\left( x^{\alpha }-\tau t^{\alpha }\right) \right) \right) \right) \right) ^{2}. \end{aligned}$$

91 $$\begin{aligned} f\left( x,t \right)= & {} \pm \frac{i}{\sqrt{\theta _3 \varrho }} \left( \pm \sqrt{\frac{\kappa }{2}}\left( \cot _{r s} \left( \sqrt{2 \kappa } ~\frac{\Gamma \left( 1+\Upsilon \right) }{\alpha }\left( x^{\alpha }-\tau t^{\alpha }\right) \right) \right. \right. \nonumber \\{} & {} \left. \left. \pm \sqrt{r s} ~\csc _{r s} \left( \sqrt{2 \kappa } ~\frac{\Gamma \left( 1+\Upsilon \right) }{\alpha }\left( x^{\alpha }-\tau t^{\alpha }\right) \right) \right) \right) \nonumber \\{} & {} \times \exp \left( \iota \frac{\Gamma \left( 1+\Upsilon \right) }{\alpha }\left( -a x^{\alpha }+\left( \frac{2 a \theta _1+\tau -a \theta _2 \tau }{\theta _2}\right) t^{\alpha }\right) \right) . \end{aligned}$$

92 $$\begin{aligned} g\left( x,t \right)= & {} 2\left( \theta _1-\theta _2 \tau \right) \left( \frac{i}{\sqrt{\theta _3 }} \left( \sqrt{\frac{\kappa }{2}}\left( \cot _{r s} \left( \sqrt{2 \kappa } ~\frac{\Gamma \left( 1+\Upsilon \right) }{\alpha }\left( x^{\alpha }-\tau t^{\alpha }\right) \right) \right. \right. \right. \nonumber \\{} & {} \left. \left. \left. \pm \sqrt{r s} ~\csc _{r s} \left( \sqrt{2 \kappa } ~\frac{\Gamma \left( 1+\Upsilon \right) }{\alpha }\left( x^{\alpha }-\tau t^{\alpha }\right) \right) \right) \right) \right) ^{2}. \end{aligned}$$

93 $$\begin{aligned} f\left( x,t \right)= & {} \pm \frac{i}{\sqrt{\theta _3 \varrho }} \left( \pm \sqrt{\frac{\kappa }{8}}\left( \tan _{r s} \left( \sqrt{\frac{\kappa }{8}} ~\frac{\Gamma \left( 1+\Upsilon \right) }{\alpha }\left( x^{\alpha }-\tau t^{\alpha }\right) \right) + \cot _{r s} \left( \sqrt{\frac{\kappa }{8}} ~\frac{\Gamma \left( 1+\Upsilon \right) }{\alpha }\left( x^{\alpha }-\tau t^{\alpha }\right) \right) \right) \right) \nonumber \\{} & {} \times \exp \left( \iota \frac{\Gamma \left( 1+\Upsilon \right) }{\alpha }\left( -a x^{\alpha }+\left( \frac{2 a \theta _1+\tau -a \theta _2 \tau }{\theta _2}\right) t^{\alpha }\right) \right) . \end{aligned}$$

94 $$\begin{aligned} g\left( x,t \right)= & {} 2\left( \theta _1-\theta _2 \tau \right) \left( \frac{i}{\sqrt{\theta _3 }} \left( \sqrt{\frac{\kappa }{8}}\left( \tan _{r s} \left( \sqrt{\frac{\kappa }{8}} ~\frac{\Gamma \left( 1+\Upsilon \right) }{\alpha }\left( x^{\alpha }-\tau t^{\alpha }\right) \right) \right. \right. \right. \nonumber \\{} & {} \left. \left. \left. + \cot _{r s} \left( \sqrt{\frac{\kappa }{8}} ~\frac{\Gamma \left( 1+\Upsilon \right) }{\alpha }\left( x^{\alpha }-\tau t^{\alpha }\right) \right) \right) \right) \right) ^{2}. \end{aligned}$$

### By generalized Kudryashov technique

For $m=1$, Eq. (32) transforms into:

95 $$\begin{aligned} H(\xi )=\alpha _{0}+\frac{\alpha _{1}}{ 1+\psi (\xi )}. \end{aligned}$$

here $\alpha _0$ and $\alpha _{1}$, are unknown constants. Inserting the Eq. (95) into Eq. (57) with Eq. (33), we get a solution set:

Set :

96 $$\begin{aligned} \left\{ \alpha _0=\pm \frac{i (\sigma -2 \Omega )}{2 \sqrt{\theta _3} \sqrt{\varrho }},\alpha _1=\pm \frac{i (\rho -\sigma +\Omega )}{\sqrt{\theta _3} \sqrt{\varrho }},\tau =\frac{\theta _1 \left( \theta _2 \left( 2 a^2+4 \rho \Omega -\sigma ^2\right) -4 a\right) }{\theta _2^2 \left( 2 a^2+4 \rho \Omega -\sigma ^2\right) -4 a \theta _2+2}\right\} . \end{aligned}$$

Case 1:

97 $$\begin{aligned} f\left( x,t\right)= & {} \pm \frac{\iota }{\sqrt{\theta _3} \sqrt{\varrho }}\left( \frac{ \left( \sigma -2 \Omega \right) }{2 }\right. \nonumber \\{} & {} \left. + \frac{ \left( \rho -\sigma +\Omega \right) }{ 1+\left( \frac{1}{2 \Omega }\left( \sqrt{4 \rho \Omega -\sigma ^2} \tan \left( \frac{1}{2} \sqrt{4 \rho \Omega -\sigma ^2} \left( d_0+\frac{\Gamma \left( 1+\Upsilon \right) }{\alpha }\left( x^{\alpha }-\tau t^{\alpha }\right) \right) \right) -\sigma \right) \right) }\right) \nonumber \\{} & {} \times \exp \left( \iota \frac{\Gamma \left( 1+\Upsilon \right) }{\alpha }\left( -a x^{\alpha }+\left( \frac{2 a \theta _1+\tau -a \theta _2 \tau }{\theta _2}\right) t^{\alpha }\right) \right) . \end{aligned}$$

98 $$\begin{aligned} g\left( x,t\right)= & {} 2\left( \theta _1-\theta _2 \tau \right) \left( \frac{\iota }{\sqrt{\theta _3} }\left( \frac{ \left( \sigma -2 \Omega \right) }{2 }\right. \right. \nonumber \\{} & {} \left. \left. + \frac{ \left( \rho -\sigma +\Omega \right) }{ 1+\left( \frac{1}{2 \Omega }\left( \sqrt{4 \rho \Omega -\sigma ^2} \tan \left( \frac{1}{2} \sqrt{4 \rho \Omega -\sigma ^2} \left( d_0+\frac{\Gamma \left( 1+\Upsilon \right) }{\alpha }\left( x^{\alpha }-\tau t^{\alpha }\right) \right) \right) -\sigma \right) \right) }\right) \right) ^{2}. \end{aligned}$$

99 $$\begin{aligned} f\left( x,t\right)= & {} \pm \frac{\iota }{\sqrt{\theta _3} \sqrt{\varrho }}\left( \frac{ \left( \sigma -2 \Omega \right) }{2 }\right. \nonumber \\{} & {} \left. + \frac{ \left( \rho -\sigma +\Omega \right) }{ 1+\left( \frac{-1}{2 \Omega }\left( \sqrt{4 \rho \Omega -\sigma ^2} \cot \left( \frac{1}{2} \sqrt{4 \rho \Omega -\sigma ^2} \left( d_0+\frac{\Gamma \left( 1+\Upsilon \right) }{\alpha }\left( x^{\alpha }-\tau t^{\alpha }\right) \right) \right) + \sigma \right) \right) }\right) \nonumber \\{} & {} \times \exp \left( \iota \frac{\Gamma \left( 1+\Upsilon \right) }{\alpha }\left( -a x^{\alpha }+\left( \frac{2 a \theta _1+\tau -a \theta _2 \tau }{\theta _2}\right) t^{\alpha }\right) \right) . \end{aligned}$$

100 $$\begin{aligned} g\left( x,t\right)= & {} 2\left( \theta _1-\theta _2 \tau \right) \left( \frac{\iota }{\sqrt{\theta _3}}\left( \frac{ \left( \sigma -2 \Omega \right) }{2 }\right. \right. \nonumber \\{} & {} \left. \left. + \frac{ \left( \rho -\sigma +\Omega \right) }{ 1+\left( \frac{-1}{2 \Omega }\left( \sqrt{4 \rho \Omega -\sigma ^2} \cot \left( \frac{1}{2} \sqrt{4 \rho \Omega -\sigma ^2} \left( d_0+\frac{\Gamma \left( 1+\Upsilon \right) }{\alpha }\left( x^{\alpha }-\tau t^{\alpha }\right) \right) \right) + \sigma \right) \right) }\right) \right) ^{2}. \end{aligned}$$

101 $$\begin{aligned} f\left( x,t\right)= & {} \pm \frac{\iota }{\sqrt{\theta _3} \sqrt{\varrho }}\left( \frac{ \left( \sigma -2 \Omega \right) }{2 }\right. \nonumber \\{} & {} \left. + \frac{ \left( \rho -\sigma +\Omega \right) }{ 1+\left( \frac{-1}{2 \Omega }\left( \sqrt{4 \rho \Omega -\sigma ^2} \tanh \left( \frac{1}{2} \sqrt{4 \rho \Omega -\sigma ^2}\left( d_0+\frac{\Gamma \left( 1+\Upsilon \right) }{\alpha }\left( x^{\alpha }-\tau t^{\alpha }\right) \right) \right) + \sigma \right) \right) }\right) \nonumber \\{} & {} \times \exp \left( \iota \frac{\Gamma \left( 1+\Upsilon \right) }{\alpha }\left( -a x^{\alpha }+\left( \frac{2 a \theta _1+\tau -a \theta _2 \tau }{\theta _2}\right) t^{\alpha }\right) \right) . \end{aligned}$$

102 $$\begin{aligned} g\left( x,t\right)= & {} \frac{-2\left( \theta _1-\theta _2 \tau \right) }{\theta _3 }\left( \frac{ \left( \sigma -2 \Omega \right) }{2 }\right. \nonumber \\{} & {} \left. + \frac{ \left( \rho -\sigma +\Omega \right) }{ 1+\left( \frac{-1}{2 \Omega }\left( \sqrt{4 \rho \Omega -\sigma ^2} \tanh \left( \frac{1}{2} \sqrt{4 \rho \Omega -\sigma ^2} \left( d_0+\frac{\Gamma \left( 1+\Upsilon \right) }{\alpha }\left( x^{\alpha }-\tau t^{\alpha }\right) \right) \right) + \sigma \right) \right) }\right) ^{2}. \end{aligned}$$

103 $$\begin{aligned} f\left( x,t\right)= & {} \pm \frac{\iota }{\sqrt{\theta _3} \sqrt{\varrho }}\left( \frac{ \left( \sigma -2 \Omega \right) }{2 }\right. \nonumber \\{} & {} \left. + \frac{ \left( \rho -\sigma +\Omega \right) }{ 1+\left( \frac{-1}{2 \Omega }\left( \sqrt{4 \rho \Omega -\sigma ^2} \coth \left( \frac{1}{2} \sqrt{4 \rho \Omega -\sigma ^2}\left( d_0+\frac{\Gamma \left( 1+\Upsilon \right) }{\alpha }\left( x^{\alpha }-\tau t^{\alpha }\right) \right) \right) + \sigma \right) \right) }\right) \nonumber \\{} & {} \times \exp \left( \iota \frac{\Gamma \left( 1+\Upsilon \right) }{\alpha }\left( -a x^{\alpha }+\left( \frac{2 a \theta _1+\tau -a \theta _2 \tau }{\theta _2}\right) t^{\alpha }\right) \right) . \end{aligned}$$

104 $$\begin{aligned} g\left( x,t\right)= & {} \frac{-2\left( \theta _1-\theta _2 \tau \right) }{\theta _3 }\left( \frac{ \left( \sigma -2 \Omega \right) }{2 }\right. \nonumber \\{} & {} \left. + \frac{ \left( \rho -\sigma +\Omega \right) }{ 1+\left( \frac{-1}{2 \Omega }\left( \sqrt{4 \rho \Omega -\sigma ^2} \coth \left( \frac{1}{2} \sqrt{4 \rho \Omega -\sigma ^2} \left( d_0+\frac{\Gamma \left( 1+\Upsilon \right) }{\alpha }\left( x^{\alpha }-\tau t^{\alpha }\right) \right) \right) + \sigma \right) \right) }\right) ^{2}. \end{aligned}$$

Case 2:

105 $$\begin{aligned} f\left( x,t\right)= & {} \pm \frac{\iota }{\sqrt{\theta _3} \sqrt{\varrho }}\left( \frac{ \left( \sigma -2 \Omega \right) }{2 }+ \frac{ \left( -\sigma +\Omega \right) }{ 1+\left( \frac{-1}{2 \Omega }\left( \sigma \tanh \left( \frac{\sigma }{2} \left( d_0+\frac{\Gamma \left( 1+\Upsilon \right) }{\alpha }\left( x^{\alpha }-\tau t^{\alpha }\right) \right) \right) + \sigma \right) \right) }\right) \nonumber \\{} & {} \times \exp \left( \iota \frac{\Gamma \left( 1+\Upsilon \right) }{\alpha }\left( -a x^{\alpha }+\left( \frac{2 a \theta _1+\tau -a \theta _2 \tau }{\theta _2}\right) t^{\alpha }\right) \right) . \end{aligned}$$

106 $$\begin{aligned} g\left( x,t\right)= & {} 2\left( \theta _1-\theta _2 \tau \right) \left( \frac{\iota }{\sqrt{\theta _3}}\left( \frac{ \left( \sigma -2 \Omega \right) }{2 }+ \frac{ \left( -\sigma +\Omega \right) }{ 1+\left( \frac{-1}{2 \Omega }\left( \sigma \tanh \left( \frac{\sigma }{2} \left( d_0+\frac{\Gamma \left( 1+\Upsilon \right) }{\alpha }\left( x^{\alpha }-\tau t^{\alpha }\right) \right) \right) + \sigma \right) \right) }\right) \right) ^{2}. \end{aligned}$$

107 $$\begin{aligned} f\left( x,t\right)= & {} \pm \frac{\iota }{\sqrt{\theta _3} \sqrt{\varrho }}\left( \frac{ \left( \sigma -2 \Omega \right) }{2 }+ \frac{ \left( -\sigma +\Omega \right) }{ 1+\left( \frac{-1}{2 \Omega }\left( \sigma \coth \left( \frac{\sigma }{2} \left( d_0+\frac{\Gamma \left( 1+\Upsilon \right) }{\alpha }\left( x^{\alpha }-\tau t^{\alpha }\right) \right) \right) + \sigma \right) \right) }\right) \nonumber \\{} & {} \times \exp \left( \iota \frac{\Gamma \left( 1+\Upsilon \right) }{\alpha }\left( -a x^{\alpha }+\left( \frac{2 a \theta _1+\tau -a \theta _2 \tau }{\theta _2}\right) t^{\alpha }\right) \right) . \end{aligned}$$

108 $$\begin{aligned} g\left( x,t\right)= & {} 2\left( \theta _1-\theta _2 \tau \right) \left( \frac{\iota }{\sqrt{\theta _3} }\left( \frac{ \left( \sigma -2 \Omega \right) }{2 }+ \frac{ \left( -\sigma +\Omega \right) }{ 1+\left( \frac{-1}{2 \Omega }\left( \sigma \coth \left( \frac{\sigma }{2} \left( d_0+\frac{\Gamma \left( 1+\Upsilon \right) }{\alpha }\left( x^{\alpha }-\tau t^{\alpha }\right) \right) \right) + \sigma \right) \right) }\right) \right) ^{2}. \end{aligned}$$

109 $$\begin{aligned} f\left( x,t\right)= & {} \pm \frac{\iota }{\sqrt{\theta _3} \sqrt{\varrho }}\left( \frac{ \left( \sigma -2 \Omega \right) }{2 }+ \frac{ \left( -\sigma +\Omega \right) }{ 1+\left( \frac{1}{2 \Omega }\left( \sqrt{-\sigma ^{2}} \tan \left( \frac{\sqrt{-\sigma ^{2}}}{2} \left( d_0+\frac{\Gamma \left( 1+\Upsilon \right) }{\alpha }\left( x^{\alpha }-\tau t^{\alpha }\right) \right) \right) - \sigma \right) \right) }\right) \nonumber \\{} & {} \times \exp \left( \iota \frac{\Gamma \left( 1+\Upsilon \right) }{\alpha }\left( -a x^{\alpha }+\left( \frac{2 a \theta _1+\tau -a \theta _2 \tau }{\theta _2}\right) t^{\alpha }\right) \right) . 
\end{aligned}$$

110 $$\begin{aligned} g\left( x,t\right)= & {} 2\left( \theta _1-\theta _2 \tau \right) \left( \frac{\iota }{\sqrt{\theta _3}}\left( \frac{ \left( \sigma -2 \Omega \right) }{2 }\right. \right. \nonumber \\{} & {} \left. \left. + \frac{ \left( -\sigma +\Omega \right) }{ 1+\left( \frac{1}{2 \Omega }\left( \sqrt{-\sigma ^{2}} \tan \left( \frac{\sqrt{-\sigma ^{2}}}{2} \left( d_0+\frac{\Gamma \left( 1+\Upsilon \right) }{\alpha }\left( x^{\alpha }-\tau t^{\alpha }\right) \right) \right) - \sigma \right) \right) }\right) \right) ^{2}. \end{aligned}$$

111 $$\begin{aligned} f\left( x,t\right)= & {} \pm \frac{\iota }{\sqrt{\theta _3} \sqrt{\varrho }}\left( \frac{ \left( \sigma -2 \Omega \right) }{2 }+ \frac{ \left( -\sigma +\Omega \right) }{ 1+\left( \frac{-1}{2 \Omega }\left( \sqrt{-\sigma ^{2}} \cot \left( \frac{\sqrt{-\sigma ^{2}}}{2} \left( d_0+\frac{\Gamma \left( 1+\Upsilon \right) }{\alpha }\left( x^{\alpha }-\tau t^{\alpha }\right) \right) \right) + \sigma \right) \right) }\right) \nonumber \\{} & {} \times \exp \left( \iota \frac{\Gamma \left( 1+\Upsilon \right) }{\alpha }\left( -a x^{\alpha }+\left( \frac{2 a \theta _1+\tau -a \theta _2 \tau }{\theta _2}\right) t^{\alpha }\right) \right) . \end{aligned}$$

112 $$\begin{aligned} g\left( x,t\right)= & {} 2\left( \theta _1-\theta _2 \tau \right) \left( \frac{\iota }{\sqrt{\theta _3}}\left( \frac{ \left( \sigma -2 \Omega \right) }{2 }\right. \right. \nonumber \\{} & {} \left. \left. + \frac{ \left( -\sigma +\Omega \right) }{ 1+\left( \frac{-1}{2 \Omega }\left( \sqrt{-\sigma ^{2}} \cot \left( \frac{\sqrt{-\sigma ^{2}}}{2} \left( d_0+\frac{\Gamma \left( 1+\Upsilon \right) }{\alpha }\left( x^{\alpha }-\tau t^{\alpha }\right) \right) \right) + \sigma \right) \right) }\right) \right) ^{2}. \end{aligned}$$

113 $$\begin{aligned} f\left( x,t\right)= & {} \pm \frac{\iota }{\sqrt{\theta _3} \sqrt{\varrho }}\left( \frac{ \left( \sigma -2 \Omega \right) }{2 }+ \frac{ \left( -\sigma +\Omega \right) }{ 1+\left( \frac{\sigma }{\sigma \exp \left( -\sigma \left( d_{0}+\frac{\Gamma \left( 1+\Upsilon \right) }{\alpha }\left( x^{\alpha }-\tau t^{\alpha }\right) \right) \right) -\Omega }\right) }\right) \nonumber \\{} & {} \times \exp \left( \iota \frac{\Gamma \left( 1+\Upsilon \right) }{\alpha }\left( -a x^{\alpha }+\left( \frac{2 a \theta _1+\tau -a \theta _2 \tau }{\theta _2}\right) t^{\alpha }\right) \right) . \end{aligned}$$

114 $$\begin{aligned} g\left( x,t\right)= & {} 2\left( \theta _1-\theta _2 \tau \right) \left( \frac{\iota }{\sqrt{\theta _3}}\left( \frac{ \left( \sigma -2 \Omega \right) }{2 }+ \frac{ \left( -\sigma +\Omega \right) }{ 1+\left( \frac{\sigma }{\sigma \exp \left( -\sigma \left( d_{0}+\frac{\Gamma \left( 1+\Upsilon \right) }{\alpha }\left( x^{\alpha }-\tau t^{\alpha }\right) \right) \right) -\Omega }\right) }\right) \right) ^{2}. \end{aligned}$$

where $$\tau =\frac{\theta _1 \left( \theta _2 \left( 2 a^2-\sigma ^2\right) -4 a\right) }{\theta _2^2 \left( 2 a^2 -\sigma ^2\right) -4 a \theta _2+2}.$$

115 $$\begin{aligned} f\left( x,t\right)= & {} \pm \frac{\iota }{\sqrt{\theta _3} \sqrt{\varrho }}\left( -\Omega + \frac{ \left( \Omega \right) }{ 1+\left( \frac{-1}{\Omega \frac{\Gamma \left( 1+\Upsilon \right) }{\alpha }\left( x^{\alpha }-\tau t^{\alpha }\right) }\right) }\right) \nonumber \\{} & {} \times \exp \left( \iota \frac{\Gamma \left( 1+\Upsilon \right) }{\alpha }\left( -a x^{\alpha }+\left( \frac{2 a \theta _1+\tau -a \theta _2 \tau }{\theta _2}\right) t^{\alpha }\right) \right) . \end{aligned}$$

116 $$\begin{aligned} g\left( x,t\right)= & {} 2\left( \theta _1-\theta _2 \tau \right) \left( \frac{\iota }{\sqrt{\theta _3}}\left( -\Omega + \frac{ \left( \Omega \right) }{ 1+\left( \frac{-1}{\Omega \frac{\Gamma \left( 1+\Upsilon \right) }{\alpha }\left( x^{\alpha }-\tau t^{\alpha }\right) }\right) }\right) \right) ^{2}. \end{aligned}$$

where $$\tau =\frac{\theta _1 \left( 2 \theta _2 a^2-4 a\right) }{2\theta _2^2 a^2 -4 a \theta _2+2}.$$

Case 3:

117 $$\begin{aligned} f\left( x,t\right)= & {} \pm \frac{\iota }{\sqrt{\theta _3} \sqrt{\varrho }}\left( \frac{ \left( -2 \Omega \right) }{2 }+ \frac{ \left( \rho +\Omega \right) }{ 1+\left( \frac{\sqrt{\rho \Omega }}{\Omega } ~\tan \left( \sqrt{\rho \Omega } \left( d_{0}+\frac{\Gamma \left( 1+\Upsilon \right) }{\alpha }\left( x^{\alpha }-\tau t^{\alpha }\right) \right) \right) \right) }\right) \nonumber \\{} & {} \times \exp \left( \iota \frac{\Gamma \left( 1+\Upsilon \right) }{\alpha }\left( -a x^{\alpha }+\left( \frac{2 a \theta _1+\tau -a \theta _2 \tau }{\theta _2}\right) t^{\alpha }\right) \right) . \end{aligned}$$

118 $$\begin{aligned} g\left( x,t\right)= & {} 2\left( \theta _1-\theta _2 \tau \right) \left( \frac{\iota }{\sqrt{\theta _3}}\left( \frac{ \left( -2 \Omega \right) }{2 }+ \frac{ \left( \rho +\Omega \right) }{ 1+\left( \frac{\sqrt{\rho \Omega }}{\Omega } ~\tan \left( \sqrt{\rho \Omega } \left( d_{0}+\frac{\Gamma \left( 1+\Upsilon \right) }{\alpha }\left( x^{\alpha }-\tau t^{\alpha }\right) \right) \right) \right) }\right) \right) ^{2}. \end{aligned}$$

119 $$\begin{aligned} f\left( x,t\right)= & {} \pm \frac{\iota }{\sqrt{\theta _3} \sqrt{\varrho }}\left( \frac{ \left( -2 \Omega \right) }{2 }+ \frac{ \left( \rho +\Omega \right) }{ 1+\left( -\frac{\sqrt{\rho \Omega }}{\Omega } ~\cot \left( \sqrt{\rho \Omega } \left( d_{0}+\frac{\Gamma \left( 1+\Upsilon \right) }{\alpha }\left( x^{\alpha }-\tau t^{\alpha }\right) \right) \right) \right) }\right) \nonumber \\{} & {} \times \exp \left( \iota \frac{\Gamma \left( 1+\Upsilon \right) }{\alpha }\left( -a x^{\alpha }+\left( \frac{2 a \theta _1+\tau -a \theta _2 \tau }{\theta _2}\right) t^{\alpha }\right) \right) . \end{aligned}$$

120 $$\begin{aligned} g\left( x,t\right)= & {} 2\left( \theta _1-\theta _2 \tau \right) \left( \frac{\iota }{\sqrt{\theta _3}}\left( \frac{ \left( -2 \Omega \right) }{2 }+ \frac{ \left( \rho +\Omega \right) }{ 1+\left( -\frac{\sqrt{\rho \Omega }}{\Omega } ~\cot \left( \sqrt{\rho \Omega } \left( d_{0}+\frac{\Gamma \left( 1+\Upsilon \right) }{\alpha }\left( x^{\alpha }-\tau t^{\alpha }\right) \right) \right) \right) }\right) \right) ^{2}. \end{aligned}$$

121 $$\begin{aligned} f\left( x,t\right)= & {} \pm \frac{\iota }{\sqrt{\theta _3} \sqrt{\varrho }}\left( \frac{ \left( -2 \Omega \right) }{2 }+ \frac{ \left( \rho +\Omega \right) }{ 1+\left( -\frac{\sqrt{\rho \Omega }}{\Omega } ~\tanh \left( \sqrt{- \rho \Omega } \left( d_{0}+\frac{\Gamma \left( 1+\Upsilon \right) }{\alpha }\left( x^{\alpha }-\tau t^{\alpha }\right) \right) \right) \right) }\right) \nonumber \\{} & {} \times \exp \left( \iota \frac{\Gamma \left( 1+\Upsilon \right) }{\alpha }\left( -a x^{\alpha }+\left( \frac{2 a \theta _1+\tau -a \theta _2 \tau }{\theta _2}\right) t^{\alpha }\right) \right) . \end{aligned}$$

122 $$\begin{aligned} g\left( x,t\right)= & {} 2\left( \theta _1-\theta _2 \tau \right) \left( \frac{\iota }{\sqrt{\theta _3} }\left( \frac{ \left( -2 \Omega \right) }{2 }+ \frac{ \left( \rho +\Omega \right) }{ 1+\left( -\frac{\sqrt{\rho \Omega }}{\Omega } ~\tanh \left( \sqrt{- \rho \Omega } \left( d_{0}+\frac{\Gamma \left( 1+\Upsilon \right) }{\alpha }\left( x^{\alpha }-\tau t^{\alpha }\right) \right) \right) \right) }\right) \right) ^{2}. \end{aligned}$$

123 $$\begin{aligned} f\left( x,t\right)= & {} \pm \frac{\iota }{\sqrt{\theta _3} \sqrt{\varrho }}\left( \frac{ \left( -2 \Omega \right) }{2 }+ \frac{ \left( \rho +\Omega \right) }{ 1+\left( -\frac{\sqrt{\rho \Omega }}{\Omega } ~\coth \left( \sqrt{- \rho \Omega } \left( d_{0}+\frac{\Gamma \left( 1+\Upsilon \right) }{\alpha }\left( x^{\alpha }-\tau t^{\alpha }\right) \right) \right) \right) }\right) \nonumber \\{} & {} \times \exp \left( \iota \frac{\Gamma \left( 1+\Upsilon \right) }{\alpha }\left( -a x^{\alpha }+\left( \frac{2 a \theta _1+\tau -a \theta _2 \tau }{\theta _2}\right) t^{\alpha }\right) \right) . \end{aligned}$$

124 $$\begin{aligned} g\left( x,t\right)= & {} 2\left( \theta _1-\theta _2 \tau \right) \left( \frac{\iota }{\sqrt{\theta _3} }\left( \frac{ \left( -2 \Omega \right) }{2 }+ \frac{ \left( \rho +\Omega \right) }{ 1+\left( -\frac{\sqrt{\rho \Omega }}{\Omega } ~\coth \left( \sqrt{- \rho \Omega } \left( d_{0}+\frac{\Gamma \left( 1+\Upsilon \right) }{\alpha }\left( x^{\alpha }-\tau t^{\alpha }\right) \right) \right) \right) }\right) \right) ^{2}. \end{aligned}$$

where $$\tau =\frac{\theta _1 (\theta _2 (2 a^2+4 \rho \Omega )-4 a)}{\theta _2^2 (2 a^2+4 \rho \Omega )-4 a \theta _2+2}.$$

125 $$\begin{aligned} f(x,t)= & {} \pm \frac{\iota }{\sqrt{\theta _3} \sqrt{\varrho }}\left( - \Omega + \frac{ \Omega }{ 1+\left( \frac{-1}{\Omega \left( d_{0}+\frac{\Gamma \left( 1+\Upsilon \right) }{\alpha }\left( x^{\alpha }-\tau t^{\alpha }\right) \right) } \right) }\right) \nonumber \\{} & {} \times \exp \left( \iota \frac{\Gamma \left( 1+\Upsilon \right) }{\alpha }\left( -a x^{\alpha }+\left( \frac{2 a \theta _1+\tau -a \theta _2 \tau }{\theta _2}\right) t^{\alpha }\right) \right) . \end{aligned}$$

126 $$\begin{aligned} g(x,t)= & {} 2\left( \theta _1-\theta _2 \tau \right) \left( \frac{\iota }{\sqrt{\theta _3} }\left( - \Omega + \frac{ \Omega }{ 1+\left( \frac{-1}{\Omega \left( d_{0}+\frac{\Gamma \left( 1+\Upsilon \right) }{\alpha }\left( x^{\alpha }-\tau t^{\alpha }\right) \right) } \right) }\right) \right) ^{2}. \end{aligned}$$

where $$\tau =\frac{\theta _1 \left( 2 \theta _2 a^2-4 a\right) }{2 \theta _2^2 a^2-4 a \theta _2+2}.$$

Case 4:

127 $$\begin{aligned} f(x,t)= & {} \pm \frac{\iota }{\sqrt{\theta _3} \sqrt{\varrho }}\left( \frac{ \left( \sigma \right) }{2 }+ \frac{ \left( \rho -\sigma \right) }{ 1+\left( \frac{1}{\sigma }\left( \exp \left( \sigma \left( d_{0}+\frac{\Gamma \left( 1+\Upsilon \right) }{\alpha }\left( x^{\alpha }-\tau t^{\alpha }\right) \right) \right) -\rho \right) \right) }\right) \nonumber \\{} & {} \times \exp \left( \iota \frac{\Gamma \left( 1+\Upsilon \right) }{\alpha }\left( -a x^{\alpha }+\left( \frac{2 a \theta _1+\tau -a \theta _2 \tau }{\theta _2}\right) t^{\alpha }\right) \right) . \end{aligned}$$

128 $$\begin{aligned} g(x,t)= & {} 2\left( \theta _1-\theta _2 \tau \right) \left( \frac{\iota }{\sqrt{\theta _3}}\left( \frac{ \left( \sigma \right) }{2 }+ \frac{ \left( \rho -\sigma \right) }{ 1+\left( \frac{1}{\sigma }\left( \exp \left( \sigma \left( d_{0}+\frac{\Gamma \left( 1+\Upsilon \right) }{\alpha }\left( x^{\alpha }-\tau t^{\alpha }\right) \right) \right) -\rho \right) \right) }\right) \right) ^{2}. \end{aligned}$$

where $$\tau =\frac{\theta _1 (\theta _2 (2 a^2 -\sigma ^2)-4 a)}{\theta _2^2 (2 a^2 -\sigma ^2)-4 a \theta _2+2}.$$

## Graphically representation

**Figure 1:**
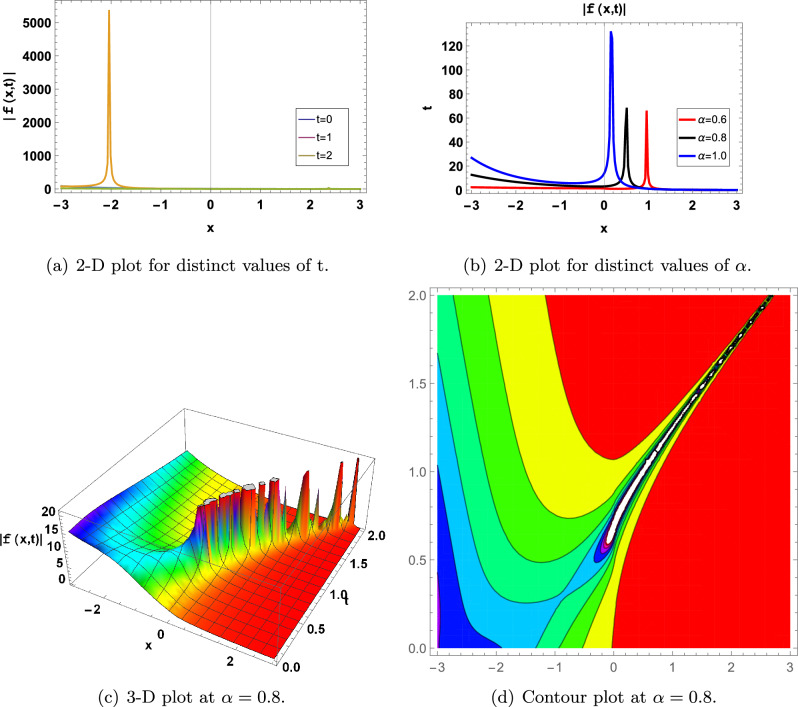
for |*f*(*x*, *t*)| represents in an Eq. (60) in two-Dimensional, three-Dimensional and contour plots at $\alpha _0=-0.01;\beta _1=0.05; d=3; \Upsilon =1; \tau =2.2; \theta _1=2; \theta _2=1;\theta _3=2; a=-1;\varrho =1$, (**a**) shows two-Dimensional for  $-3<x<3$ when $\alpha =1$, Blue graph when $t=0$, Orange graph when $t=1$, Green graph when $t=2$, (**b**) shows the 2-D plot for $-3<x<3$ and $0<t<2$, where the Red line when $\alpha =0.6$, the Black line when $\alpha =0.8$, and the Blue line when $\alpha =1$, (**c**) shows three-Dimensional and (**d**) shows contour plot when $\alpha =0.8$ and $0<t$ and $t<2$.

**Figure 2:**
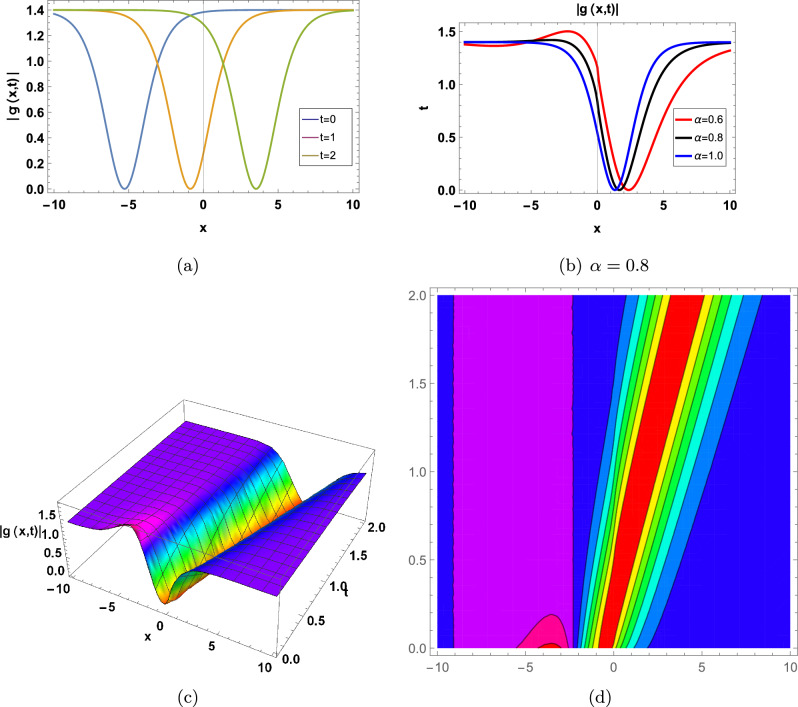
for |*g*(*x*, *t*)| represents in an Eq. (64) in two-Dimensional, three-Dimensional and contour plots at $\alpha _0=-0.5;\beta _1=0.7; d=3; \Upsilon =1; \tau =2.2; \theta _1=2; \theta _2=1;\theta _3=2; a=-1;\varrho =1$ (**a**) shows two-Dimensional for  $-10<x<10$ when $\alpha =1$, Blue graph when $t=0$, Orange graph when $t=1$, Green graph when $t=2$, (**b**) shows the 2-D plot for $-10<x<10$ and $0<t<2$, where the Red line when $\alpha =0.6$, the Black line when $\alpha =0.8$, and the Blue line when $\alpha =1$, (**c**) shows three-Dimensional and (**d**) shows contour plot when $\alpha =0.8$ and $0<t$ and $t<2$.

**Figure 3:**
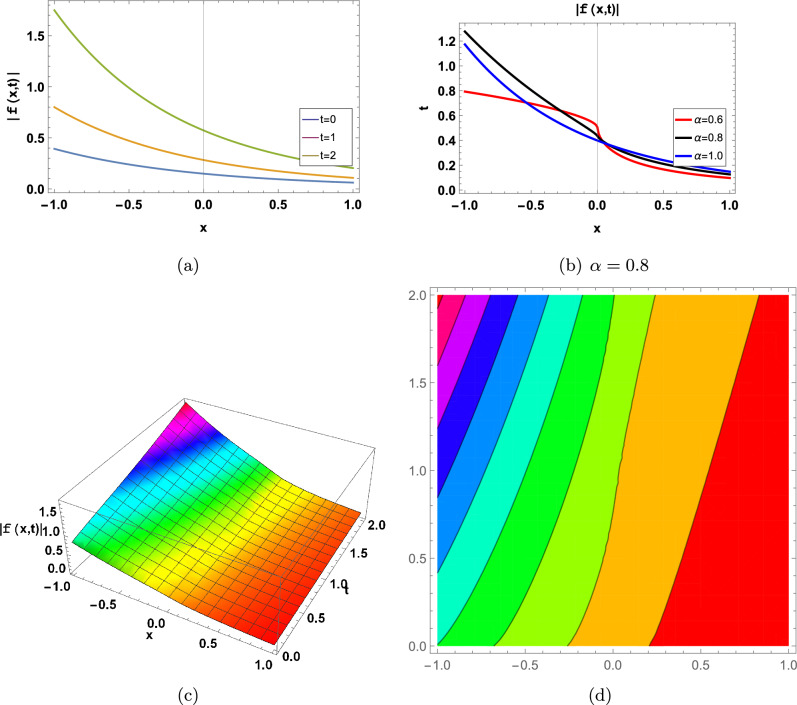
for |*f*(*x*, *t*)| represents in an Eq. (71) in two-Dimensional, three-Dimensional and contour plots at $\kappa =-0.08;\Upsilon =1;\theta _1=1;\theta _2=2;\theta _3=1; a=-1;\varrho =1$ (**a**) shows two-Dimensional for  $-1<x<1$ when $\alpha =1$, Blue graph when $t=0$, Orange graph when $t=1$, Green graph when $t=2$, (**b**) shows the 2-D plot for $-1<x<1$ and $0<t<2$, where the Red line when $\alpha =0.6$, the Black line when $\alpha =0.8$, and the Blue line when $\alpha =1$, (**c**) shows three-Dimensional and (**d**) shows contour plot when $\alpha =0.8$ and $0<t$ and $t<2$.

**Figure 4:**
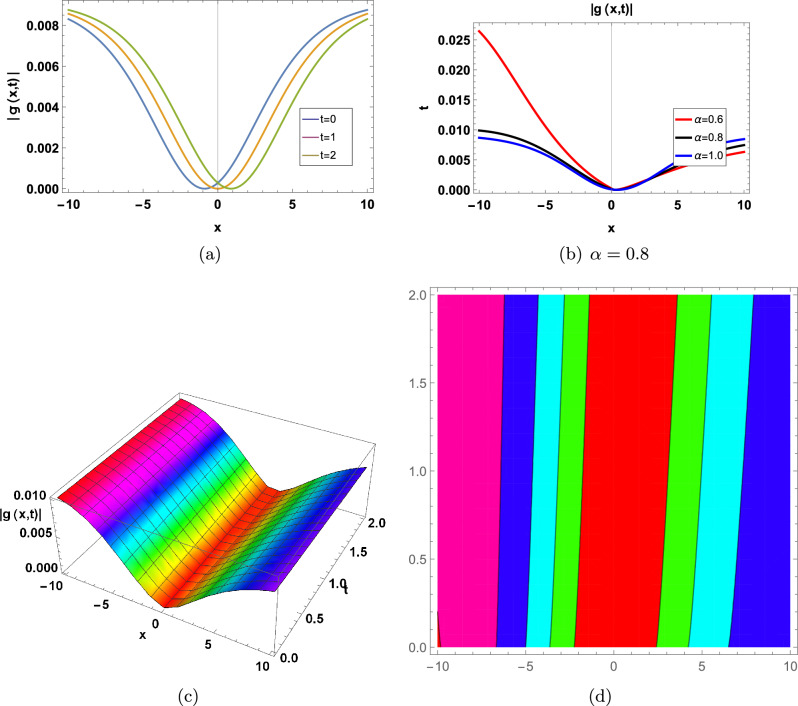
for |*g*(*x*, *t*)| represents in an Eq. (76) in two-Dimensional, three-Dimensional and contour plots at $\kappa =-0.08;\Upsilon =1;\theta _1=1;\theta _2=2;\theta _3=1; a=-1$ (**a**) shows two-Dimensional for  $-10<x<10$ when $\alpha =1$, Blue graph when $t=0$, Orange graph when $t=1$, Green graph when $t=2$, (**b**) shows the 2-D plot for $-10<x<10$ and $0<t<2$, where the Red line when $\alpha =0.6$, the Black line when $\alpha =0.8$, and the Blue line when $\alpha =1$, (**c**) shows three-Dimensional and (**d**) shows contour plot when $\alpha =0.8$ and $0<t$ and $t<2$.

**Figure 5:**
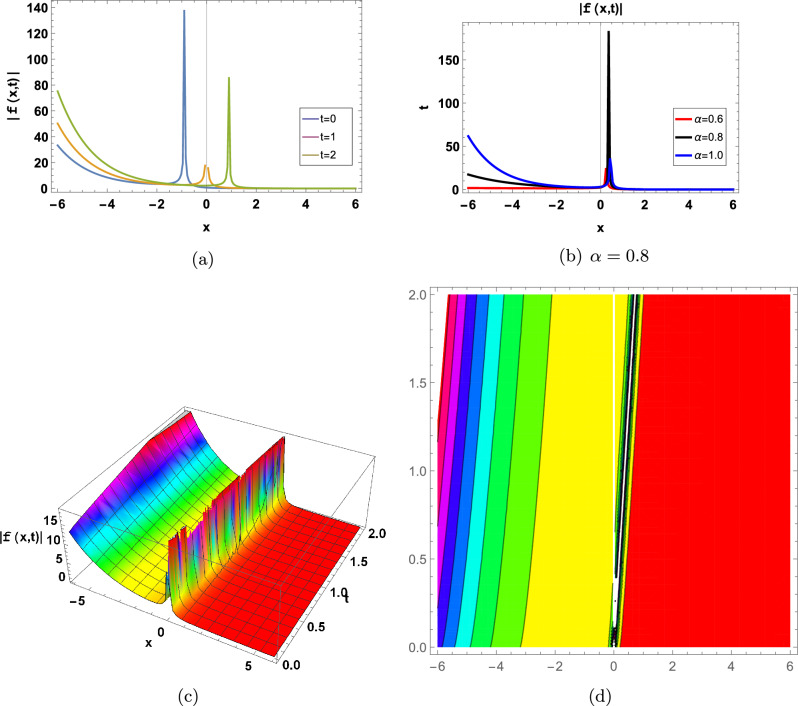
for |*f*(*x*, *t*)| represents in an Eq. (85) in two-Dimensional, three-Dimensional and contour plots at $\kappa =0.04;\Upsilon =1;\theta _1=1;\theta _2=2;\theta _3=1; a=-1;\varrho =1$ (**a**) shows two-Dimensional for  $-6<x<6$ when $\alpha =1$, Blue graph when $t=0$, Orange graph when $t=1$, Green graph when $t=2$, (**b**) shows the 2-D plot for $-6<x<6$ and $0<t<2$, where the Red line when $\alpha =0.6$, the Black line when $\alpha =0.8$, and the Blue line when $\alpha =1$, (**c**) shows three-Dimensional and (**d**) shows contour plot when $\alpha =0.8$ and $0<t$ and $t<2$.

**Figure 6:**
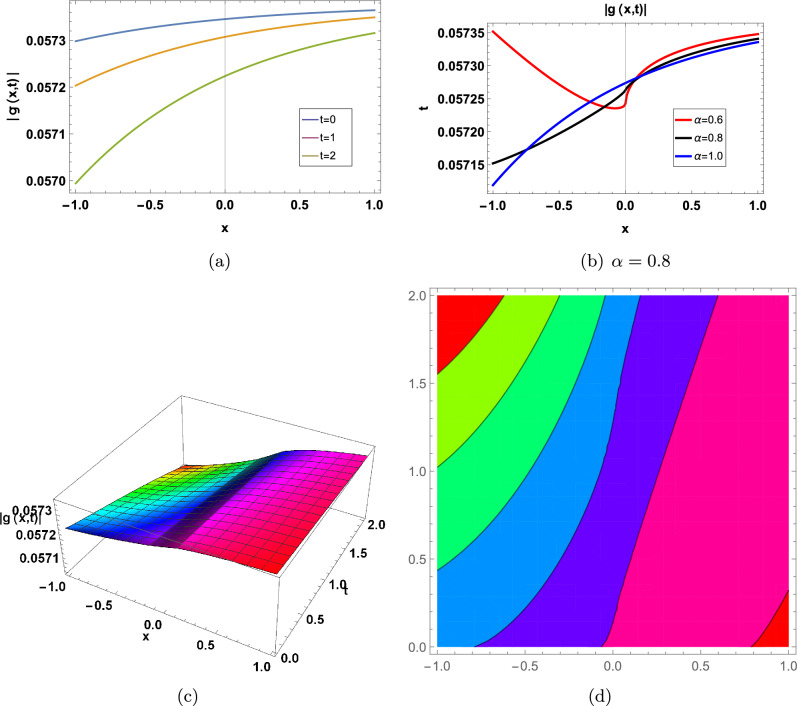
for |*g*(*x*, *t*)| represents in an Eq. (98) in two-Dimensional, three-Dimensional and contour plots at $\Omega =-0.05;\Upsilon =1;\theta _1=1;\theta _2=2;\theta _3=1; a=-0.5;\varrho =1;\sigma =0.2;d_0=1$ (**a**) shows two-Dimensional for  $-20<x<20$ when $\alpha =1$, Blue graph when $t=0$, Orange graph when $t=1$, Green graph when $t=2$, (**b**) shows the 2-D plot for $-20<x<20$ and $0<t<2$, where the Red line when $\alpha =0.6$, the Black line when $\alpha =0.8$, and the Blue line when $\alpha =1$, (**c**) shows three-Dimensional and (**d**) shows contour plot when $\alpha =0.8$ and $0<t$ and $t<2$7.

**Figure 7:**
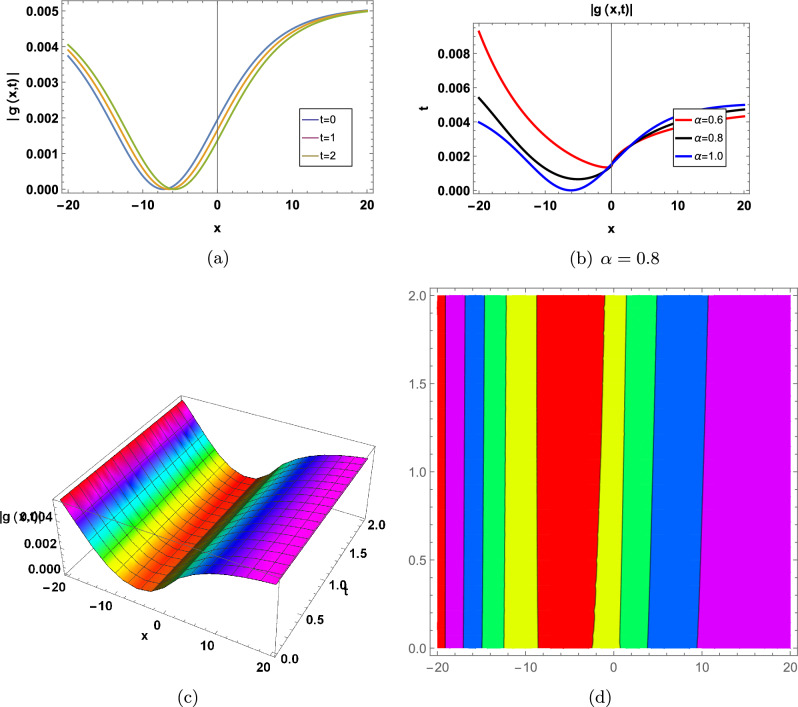
for |*g*(*x*, *t*)| represents in an Eq. (112) in two-Dimensional, three-Dimensional and contour plots at $\kappa =-0.08;\Upsilon =1;\theta _1=1;\theta _2=2;\theta _3=1; a=-1;\varrho =1$ (**a**) shows two-Dimensional for  $-1<x<1$ when $\alpha =1$, Blue graph when $t=0$, Orange graph when $t=1$, Green graph when $t=2$, (**b**) shows the 2-D plot for $-1<x<1$ and $0<t<2$, where the Red line when $\alpha =0.6$, the Black line when $\alpha =0.8$, and the Blue line when $\alpha =1$, (**c**) shows three-Dimensional and (**d**) shows contour plot when $\alpha =0.8$ and $0<t$ and $t<2$7.

Some of the above achieved results are shown graphically in the following (Figs. 1, 2, 3, 4, 5, 6 and 7).

## Results and discussion

In this section, the obtained solutions and the earlier existing results are compared. In^14^, breather wave, rogue wave and semi-rational solutions of have been obtained by using the J-fold Darboux transformation. In^28^ Jacobi elliptic function solutions have been achieved by utilizing the new extended auxiliary equation technique. But we considered the Akbota equation in the sense of truncated M-fractional derivative to gain the newer and very close results to the numerical solutions. We used the techniques that are provide the distinct types of solutions such as periodic, singular, bright–dark, kink and others.

## Conclusion

We are succeed to attain the novel analytical soliton solutions to the nonlinear $(1+1)$-dimensional Akbota equation with truncated M-fractional derivative. For this purpose, we utilize the $\exp _a$ function, Sardar sub-equation and generalized kudryashov techniques. Concerning model, as a Heisenberg ferromagnetic kind model, have much importance in the various nonlinear phenomenon. Few of the achieved results are shown by 2-D, 3-D and contour plots in Figs. 1, 2, 3, 4, 5, 6 and 7. Fractional effect on solutions are also shown in graphs. The obtained results are very helpful in optical fibers, optics, telecommunications and other fields. Hence, the gained solutions are fruitful in the future study for these models. The used techniques provide the different variety of solutions.Our gained solutions will be helpful in the further study of the concerned model.

## Data Availability

The datasets used and/or analysed during the current study available from the corresponding author on reasonable request.
